# Using the mood disorder questionnaire and bipolar spectrum diagnostic scale to detect bipolar disorder and borderline personality disorder among eating disorder patients

**DOI:** 10.1186/1471-244X-13-69

**Published:** 2013-02-25

**Authors:** Toshihiko Nagata, Hisashi Yamada, Alan R Teo, Chiho Yoshimura, Yuya Kodama, Irene van Vliet

**Affiliations:** 1 Department of Neuropsychiatry, Osaka City University Graduate School of Medicine, 1-4-3 Asahimachi, Abunoku, Osaka 545-8585, Japan; 2 Department of Neuropsychiatry, Hyogo College of Medicine, Nishinomiya, Japan; 3 Department of Internal Medicine and Department of Psychiatry, University of Michigan, Ann Arbor, USA; 4 Department of Psychiatry, Leiden University Medical Center, Leiden, the Netherlands

**Keywords:** Bipolar disorder, Borderline personality disorder, Eating disorder, Comorbidity, Screening scale

## Abstract

**Background:**

Screening scales for bipolar disorder including the Mood Disorder Questionnaire (MDQ) and Bipolar Spectrum Diagnostic Scale (BSDS) have been plagued by high false positive rates confounded by presence of borderline personality disorder. This study examined the accuracy of these scales for detecting bipolar disorder among patients referred for eating disorders and explored the possibility of simultaneous assessment of co-morbid borderline personality disorder.

**Methods:**

Participants were 78 consecutive female patients who were referred for evaluation of an eating disorder. All participants completed the mood and eating disorder sections of the SCID-I/P and the borderline personality disorder section of the SCID-II, in addition to the MDQ and BSDS. Predictive validity of the MDQ and BSDS was evaluated by Receiver Operating Characteristic analysis of the Area Under the Curve (AUC).

**Results:**

Fifteen (19%) and twelve (15%) patients fulfilled criteria for bipolar II disorder and borderline personality disorder, respectively. The AUCs for bipolar II disorder were 0.78 (MDQ) and 0.78 (BDSD), and the AUCs for borderline personality disorder were 0.75 (MDQ) and 0.79 (BSDS).

**Conclusions:**

Among patients being evaluated for eating disorders, the MDQ and BSDS show promise as screening questionnaires for both bipolar disorder and borderline personality disorder.

## Background

As bipolar disorders are serious mental disorders that can cause severe lifelong functional impairment, early recognition of the diagnosis and early introduction of mood stabilizers are crucial for improvement of outcomes [1]. Nonetheless, most patients with bipolar disorder go years before receiving an appropriate diagnosis and starting mood stabilizers [1]. Borderline personality disorder is the most common personality disorder in clinical settings, and causes marked distress and impairment in social, occupational, and role functioning [2]. Yet, similar to bipolar disorder, borderline personality disorder is often incorrectly diagnosed or underdiagnosed in clinical practice [2]. Both bipolar and borderline personality disorders are associated with high rates of completed suicide [1,2] and are common among patients with mood disorders [1,2] and eating disorders [3,4].

Recently, the boundary of these disorders has been a focus of debate [5-9]. There are two viewpoints about the relationship between bipolar disorder, especially bipolar II disorder, and borderline personality disorder. The first one is that underlying cyclothymic temperament can explain the relationship, and borderline personality disorder [9] (as well as bulimia nervosa [8]) are variants of bipolar disorders. Others insist that there is clear boundary between bipolarity and borderline personality disorder, and they resist the expansion of bipolar disorder as an invasion of “bipolar imperialism” toward other diagnostic categories [5].

Two screening scales, the Mood Disorder Questionnaire (MDQ) [10] and Bipolar Spectrum Diagnostic Scale (BSDS) [11], have been developed to ameliorate the underdiagnosis of bipolar disorders. These instruments show good psychometric properties to detect bipolarity among patients with unipolar depression and are recommended as screening tools for bipolar disorders among patients with unipolar depression [1]. However, Zimmerman et al. (2011) [12,13] reported that the false positive rates of these two scales were not negligible because of the symptomatic overlap of bipolar disorder and other conditions [1]. Patients diagnosed with bipolar disorder by previous doctors were significantly more likely to be diagnosed with borderline personality disorder compared to patients who were not diagnosed with bipolar disorder (24.4% vs. 6.1%) [14]. Viewed another way, these results suggest the possibility that both bipolar and borderline personality disorders can be simultaneously detected by these scales.

To the best of our knowledge, the MDQ and BSDS have never been used to detect bipolar disorders or borderline personality disorder among eating disorder patients, despite the relatively high comorbidity rates of these disorders [3,4]. In contrast to common attention towards impulsivity and borderline personality disorder among eating disorder patients [4], the presence of comorbid bipolar disorder has rarely received attention of eating disorder specialists [15]. However, some evidence suggests increased prevalence of bipolar II disorder [16], ego-syntonic hypomania may escape clinical detection, and comorbid bipolar disorder requires special therapeutic considerations [3]. Thus, screening scales for bipolar disorder might be more important than eating disorder specialists traditionally thought.

The aim of the current study was to examine the diagnostic accuracy (including sensitivity and specificity) of the MDQ and BSDS to detect bipolar disorders among patients that were referred for evaluation of an eating disorder. We also explored the possibility that the two scales can detect borderline personality disorder among this population. We hypothesized that the diagnostic accuracy of the two screening tests (MDQ and BSDS) for borderline personality disorder might be similar to that for bipolar disorders.

## Methods

### Participants

Participants were recruited from a consecutive series of ninety female patients who were referred for evaluation and/or treatment of an eating disorder to the first and second authors (T. N. and H. Y.) at the Department of Neuropsychiatry, Osaka City University Hospital from February to June 2011. To maximize real world applicability of findings, exclusion criteria were minimal: 1) substance use disorder requiring acute detoxification (if such cases hospital receptionists recommended patients to visit an alcohol treatment facility), 2) self-reported history of schizophrenia, schizoaffective disorder, schizophreniform disorder, or organic mental illnesses, as determined by the screening questionnaire of the SCID-I/P, or 3) inability or unwillingness to complete self-rating scales. The patients with eating disorder not otherwise specified (EDNOS) were not excluded as prior research has shown these patients often are referred for eating disorder treatment and may have comorbid bipolar or borderline personality disorder [17] . Of the ninety patients who were screened, seventy-eight patients enrolled in the study, and all enrollees completed the study. Of the twelve patients that did not participate in the study, six deemed their psychiatric problem minor and opted to cope with the difficulty, four were reluctant to undergo detailed psychiatric assessment, and two declined to participate for unknown reasons. As part of routine clinical care, patients received cognitive behavioral therapy, dialectical behavioral therapy, or medication management depending on the results of their assessment, even when their provisional primary diagnosis (defined as the disorder most influencing their global functioning) was other than an eating disorder. All patients provided written informed consent before entering the study. This study was approved by the institutional review committee of the Osaka City University Graduate School of Medicine.

### Measurements

Two self-report screening scales for bipolar disorder, the Mood Disorder Questionnaire (MDQ) [10,18] and Bipolar Spectrum Diagnostic Scale (BSDS) [11,19], were competed by all participants. The MDQ screens for a lifetime history of mania or hypomania using 13 dichotomous (yes/no) symptom questions reflecting the DSM-IV inclusion criteria. The symptom questions are followed by a single question about whether the symptoms clustered during the same period. The final question evaluates the level of impairment resulting from the symptoms on a 4-point scale (no, minor, moderate, or serious problems). A score of 7 or more on the first 13 items, “yes” to symptom clustering, and moderate or greater problems was proposed as the cut-off level in the original study [10]. In a Japanese study of unipolar depressive patients, a lower cut-off of more than 5 with minor or greater problems was proposed [19]. The BSDS was developed to target bipolar II disorder and bipolar disorder not otherwise specified and supplement clinicians’ semi-structured interviews [11]. The BSDS consists of two parts: first, a paragraph containing 19 statements describing many of the symptoms of bipolar disorder, and, second, a single multiple-choice question asking respondents how well the paragraph describes them. The total score ranges from 0 to 25. A score of 13 for the original version [11], 12 for the Chinese version [20], and 11 for the Japanese version [19] have been proposed as cut-off points.

All participants underwent a direct (face-to-face) assessment conducted by T. N. or H. Y. who each have more than ten years’ experience treating eating disorders. This assessment included the mood and eating disorder sections of the Structured Clinical Interview for DSM-IV, (SCID-I/P) [21,22], and the borderline and histrionic personality sections of the Structured Clinical Interview for DSM-IV Personality Disorders (SCID-II) [23,24]. These limited portions of the SCID-II were selected because our previous study found that the prevalence of other personality disorders such as antisocial and narcissistic personality disorder were low (0 and 3%, respectively) in our setting [25] while previous studies [7,26] have suggested a relationship between bipolarity and histrionic personality disorder.

### Statistical analysis

We examined the diagnostic accuracy of the MDQ and BSDS for bipolar as well as personality disorders, using the SCID-I and II as the gold standard diagnostic tool. Sensitivity, specificity and likelihood ratio for a positive test [LR+, sensitivity/(1-specificity)] [27] were calculated according to several cut-off points suggested by previous studies [10,11,18-20]. To the best of our knowledge, no previous study has explored the possibility that the two scales might detect borderline or histrionic personality disorders. Given this, we used the same cut-off point to detect the personality disorders as for bipolar disorder. In addition, the Receiver Operating Characteristic (ROC) curve and Area Under the Curve (AUC) of both scales for bipolar and personality disorders were calculated. AUC is a preferred measure of accuracy as it is uninfluenced by prevalence, which would be expected to be higher in this study’s patient population. All data were analyzed with SPSS 16.0 (SPSS, Inc., Chicago in USA).

## Results

The patients’ demographic and clinical characteristics are depicted in Table 1. A high level of functional impairment was suggested by the high rate of single participants (around two-thirds), unemployment (around half) and chronicity (around ten years).

**Table 1:** Demographic and clinical characteristics of participants (n = 78) with eating disorders

	**Mean (SD) or N (%)**
Age, years	29.5 (7.4)
Education, years	13.8 (2.4)
Marital status, single	58 (74%)
Occupational status	
Unemployed	37 (47%)
Part-time worker (or student)	16 (21%)
Full-time worker (or student)	25 (32%)
Age at onset of eating disorder	19.1 (4.8)
Body mass index	17.3 (4.4)
Subtype of eating disorder	
Anorexia nervosa restricting type	11 (14%)
Anorexia nervosa binge-eating purging subtype	24 (31%)
Bulimia nervosa purging subtype	27 (35%)
Restricting EDNOS	2 (3%)
Binge-eating / purging EDNOS	14 (18%)
Frequency of binge eating (episodes/week)	4.6 (4.6)
Frequency of vomiting (episodes/week)	4.9 (4.2)
History of major depressive episode(s)	55 (71%)
Age at onset, years	20.5 (4.8)
History of manic episode(s)	0 (0%)
History of hypomanic episode(s)	15 (19%)
Age at onset, years	23.1 (5.6)
Borderline personality disorder	12 (15%)
Histrionic personality disorder	23 (30%)
BSDS total score	9.2 (6.2)
BSDS≥13	22 (28%)
BSDS≥11	31 (40%)
MDQ≥7 with moderate functional impairment	15 (19%)
MDQ≥5 with minor functional impairment	30 (39%)

*MDQ*: Mood Disorder Questionnaire, *BSDS*: Bipolar Spectrum Diagnostic Scale, *EDNOS*: Eating disorder not otherwise specified.

Fifty-five (71%) had a lifetime history of a major depressive episode and 15 (19%) a hypomanic episode (bipolar II disorder). No patients had bipolar I disorder. Twelve (15%) had borderline personality disorder and 23 (29%) histrionic personality disorder. Five (6%) had both lifetime bipolar II disorder and borderline personality disorder. Similarly, eight (10%) had both lifetime bipolar II disorder and histrionic personality disorder. No patients with anorexia nervosa restricting subtype or restricting EDNOS had bipolar, borderline, or histrionic personality disorders.

**Figure 1:**
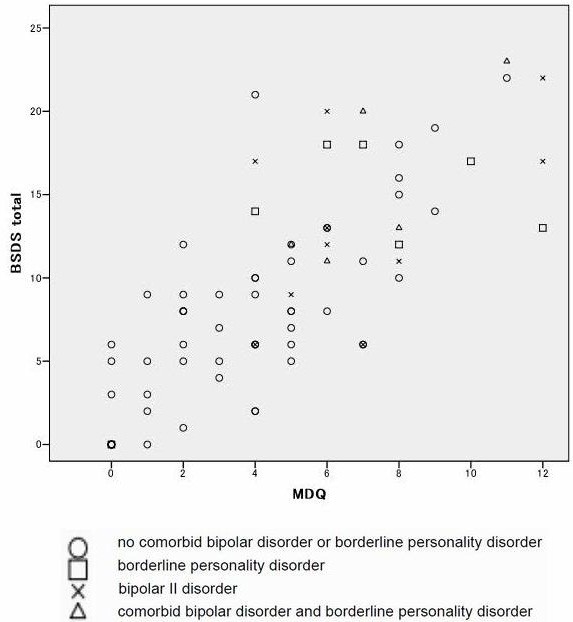
**Diagnoses of patients (n=78) based on MDQ and BSDS scores.** MDQ: Mood Disorder Questionnaire, BSDS: Bipolar Spectrum Diagnostic Scale.

Figure 1 shows the monomodal distribution (rather than bimodal) of patients’ score on the MDQ and BSDS. As Table 2 shows, both the MDQ and BSDS exhibited similar sensitivity, specificity, and likelihood ratios (LR+) for detecting bipolar II disorder. Further, these two scales showed comparable accuracy in detecting borderline personality disorder. Since accuracy for detecting histrionic personality disorder was relatively low, just bipolar II disorder and borderline personality disorder were the focus of the following analyses. Results were similar for detecting bipolar II disorder without comorbid borderline personality disorder and borderline personality disorder without comorbid bipolar II disorder.

**Table 2:** Sensitivity and specificity of the MDQ and BSDS to diagnose bipolar disorder, borderline personality disorder, and histrionic personality disorder according to several cut-off points

	**BPII**	**BPD**	**Histrionic**
	N (sensitivity, specificity, LR+)		
	15	12	23
MDQ≥7 with moderate functional impairment	3 (0.20, 0.81, 1.1)	5 (0.42, 0.85, 2.8)	6 (0.26, 0.84, 1.6)
MDQ≥6 with moderate functional impairment	8 (0.53, 0.78, 2.4)	7 (0.58, 0.80, 2.9)	7 (0.30, 0.76, 2.1)
MDQ≥6 with minor functional impairment	8 (0.53, 0.78, 2.4)	7 (0.58, 0.77, 2.5)	8 (0.35, 0.75, 1.4)
MDQ≥5 with minor functional impairment	10 (0.67, 0.68, 2.1)	8 (0.67, 0.67, 2.0)	12 (0.52, 0.67, 1.6)
BSDS≥13	8 (0.53, 0.78, 2.4)	8 (0.67, 0.79, 3.2)	10 (0.44, 0.78, 2.0)
BSDS≥12	10 (0.67, 0.73, 2.9)	10 (0.83, 0.74, 3.2)	12 (0.52, 0.73, 1.9)
BSDS≥11	12 (0.80, 0.70, 2.7)	11 (0.92, 0.70, 3.1)	15 (0.65, 0.71, 2.2)
BSDS≥10	12 (0.80, 0.64, 2.2)	11 (0.92, 0.64, 2.6)	15 (0.65, 0.64, 1.8)
BSDS≥9	13 (0.88, 0.59, 2.2)	11(0.92, 0.58, 2.2)	18 (0.78, 0.82, 4.3)
	BPII only	BPD only	
	N (sensitivity, specificity, LR+)		
	10	7	
MDQ≥7 with moderate functional impairment	2 (0.20, 0.81, 1.1)	4 (0.57, 0.78, 2.6)	
MDQ≥5 with minor functional impairment	7 (0.70,0.66, 2.1)	5 (0.71, 0.65, 2.0)	
BSDS≥13	5 (0.50, 0.75, 2.0)	5 (0.71, 0.70, 2.4)	
BSDS≥12	6 (0.60, 0.69, 1.9)	6 (0.86, 0.70, 2.9)	
BSDS≥11	7 (0.70, 0.67, 2.1)	6 (0.86, 0.65, 2.5)	

*MDQ*: Mood Disorder Questionnaire, *BSDS*: Bipolar Spectrum Diagnostic Scale, *BPII*: bipolar II disorder, *BPII* only: bipolar II disorder without borderline personality disorder, *BPD*: borderline personality disorder, *BPD* only: borderline personality disorder without bipolar II disorder, Histrionic; Histrionic personality disorder, *LR*+: Likelihood Ratio for a positive test.

**Figure 2:**
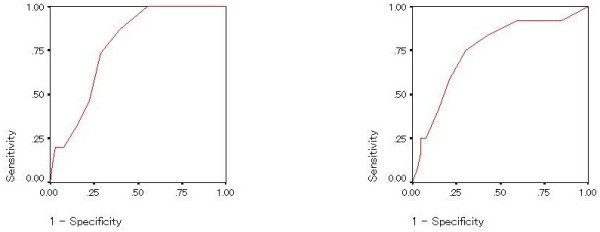
Receiver Operating Characteristic (ROC) curve of diagnostic accuracy of score on question one of the mood disorder questionnaire; Bipolar II disorder (left) and borderline personality disorder (right).

**Figure 3:**
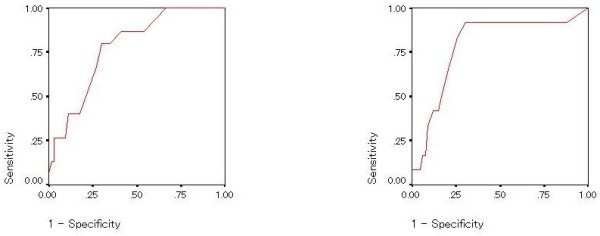
Receiver Operating Characteristic (ROC) curve of diagnostic accuracy of score on the mood disorder questionnaire; Bipolar II disorder without borderline personality disorder (left) and borderline personality disorder without bipolar II disorder (right).

To evaluate the ability to detect bipolar II disorders, ROC curves and the AUCs (95% Confidence Interval) of the MDQ (Figure 2) and BSDS (Figure 3) were calculated. The AUC of the MDQ was determined using the total score of part one because using level of impairment data would require multiple ROC curves [13].

The AUCs for the MDQ and BSDS in detecting bipolar II disorder were 0.779 (0.674-0.885) and 0.778 (0.662-0.895), respectively (Figures 2 and 3).

**Figure 4:**
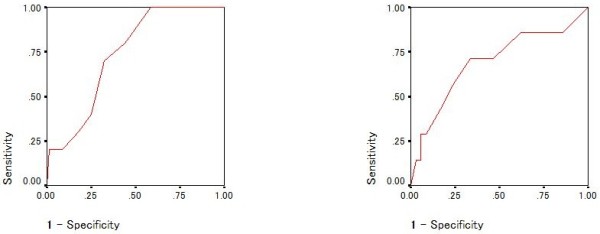
Receiver Operating Characteristic (ROC) curve of diagnostic accuracy of score on question one of the bipolar spectrum diagnostic scale; Bipolar II disorder (left) and borderline personality disorder (right).

**Figure 5:**
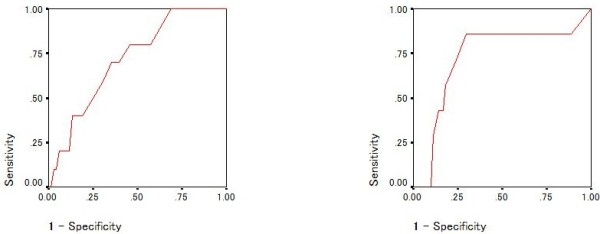
Receiver Operating Characteristic (ROC) curve of diagnostic accuracy of score on the bipolar spectrum diagnostic scale; Bipolar II disorder without borderline personality disorder (left) and borderline personality disorder without bipolar II disorder (right).

Similarly, to explore the ability to detect borderline personality disorder with or without co-morbid bipolar II disorder, ROC curves and the AUC (95% Confidence Interval) of the MDQ (Figures 2 and 4) and BSDS (Figures 3 and 5) were calculated. In detecting borderline personality, the AUCs for the MDQ and BSDS disorder were 0.750 (0.597-0.903) and 0.787 (0.640-0.934), respectively (Figures 2 and 3). In order to control for the effect of co-morbidity accounting for the diagnostic accuracy of the scales, ROC curves were also calculated for patients with bipolar II disorder but not borderline personality disorder and vice versa. Results remained statistically significant except for the MDQ detecting borderline personality disorder without comorbid bipolar II disorder. Specifically, the AUC for the MDQ was 0.737 (0.605-0.868) for the bipolar II disorder but not borderline personality disorder and 0.691 (0.460-0.923) for borderline personality disorder without comorbid bipolar II disorder. For the BSDS, the AUC was 0.715 (0.566-0.865) for bipolar II disorder but not borderline personality disorder and 0.723 (0.506-0.940) for borderline personality disorder without comorbid bipolar II disorder.

## Discussion

Prior research has suggested that the MDQ and BSDS are useful instruments to detect bipolar disorders among patients with recurrent depressive episodes [10,11]. The current study expands on this work by showing that these two scales can detect bipolar disorder among patients with eating disorders. Both the MDQ and BSDS screening scales exhibited similar value in terms of predictive validity in a population of patients presenting to a psychiatric clinic in a tertiary-care setting. Thus, the MDQ and BSDS offer reasonably comparable diagnostic accuracy in the form of a self-report measure that requires much less time, expertise, and resources to administer than the SCID or Composite International Diagnostic Interview (CIDI). By comparison, the AUC was 0.834 for concordance of bipolar II disorder diagnosis between the SCID and CIDI in a community population [28].

In addition, this study suggests that bipolar disorder and borderline personality disorder can both be detected with moderate accuracy by use of a brief screening instrument, although it was difficult for these scales to detect histrionic personality disorder. Results were similar even after controlling for co-morbidity. This study importantly shows that the two scales can be used as screening tools for borderline personality disorder in a real world setting where eating disorder specialists work. In addition, results showed the assessment of affective instability is useful in terms of bipolar and borderline personality disorder [3], although impulsivity has traditionally been focused in the eating disorder field [4].

Accurate diagnosis and distinction of bipolar disorder and borderline personality disorder is important because of the differing treatment approaches. Psychotherapeutic approaches for these two disorders are very different [1,4,29]. Also, pharmacotherapy is a core component of treatment for bipolar disorder, but only adjunctive and symptom-targeted for borderline personality disorder [1,29]. These two scales are useful to detect cases that require careful assessment before starting antidepressants, although these scales cannot differentiate between bipolarity and borderline personality disorder.

There are a number of important limitations regarding this study. Sensitivity of these two scales may not be considered sufficiently high using the cut-off point that the original studies suggested. It remains to be known whether the lower sensitivity is due to differences of culture, population, or clinical setting where the participants were recruited. Results are from a single treatment center, and males and patients with bipolar I disorder were not included. Results were not analyzed in relationship with eating disorder subtypes (such as restricting or binge/purging) due to a modest sample size. Assessment was cross-sectional, and careful longitudinal consideration is essential because patients with either bipolar or borderline personality disorder can present with similar symptomatology at a given time [6]. This is especially true in the case of comorbid eating disorder because chaotic eating behaviors and starvation might influence symptomatology [30,31] including dysphoria [32] and anger [9].

## Conclusions

Nonetheless, current results show that these two scales (MDQ and BSDS) are helpful to detect cases that need careful assessment. In addition, we believe these results should encourage further attempts to reconsider the relationship between and symptomatology of bipolar disorder and borderline personality disorder.

## Abbreviations

MDQ: Mood Disorder Questionnaire;BSDS: Bipolar Spectrum Diagnostic Scale;EDNOS: Eating disorder not otherwise specified;SCID-I/P: Structured Clinical Interview for DSM-IV;SCID-II: Structured Clinical Interview for DSM-IV Personality Disorders;LR+: Likelihood ratio for a positive test;ROC: Receiver Operating Characteristic;AUC: Area Under the Curve;CIDI: Composite International Diagnostic Interview

## Competing interests

All authors declare that there is no conflict of interest.

## Authors’ contribution

All authors contributed to the design of this study. TN and AT drafted the manuscript. All authors contributed to revision of the manuscript. All authors read and approved the final manuscript.

## Pre-publication history

The pre-publication history for this paper can be accessed here:

http://www.biomedcentral.com/1471-244X/13/69/prepub
